# Neuropsychological profile according to the clinical stage of young persons presenting for mental health care

**DOI:** 10.1186/2050-7283-1-8

**Published:** 2013-05-14

**Authors:** Daniel F Hermens, Sharon L Naismith, Jim Lagopoulos, Rico S C Lee, Adam J Guastella, Elizabeth M Scott, Ian B Hickie

**Affiliations:** Clinical Research Unit, Brain and Mind Research Institute, University of Sydney, 100 Mallet Street, Camperdown, NSW 2050 Australia

**Keywords:** Neuropsychology, Clinical staging, Psychiatric, Young adults

## Abstract

**Background:**

Clinical staging of mental disorders proposes that individuals can be assessed at various sub-syndromal and later developed phases of illness. As an adjunctive rating, it may complement traditional diagnostic silo-based approaches. In this study, we sought to determine the relationships between clinical stage and neuropsychological profile in young persons presenting to youth-focused mental health services.

**Methods:**

Neuropsychological testing of 194 help-seeking young people (mean age 22.6 years, 52% female) and 50 healthy controls. Clinical staging rated 94 persons as having an ‘attenuated syndrome’ (stage 1b) and 100 with a discrete or persistent disorder (stage 2/3).

**Results:**

The discrete disorder group (stage 2/3) showed the most impaired neuropsychological profile, with the earlier stage (1b) group showing an intermediate profile, compared to controls. Greatest impairments were seen in verbal memory and executive functioning. To address potential confounds created by ‘diagnosis’, profiles for those with a mood syndrome or disorder but not psychosis were also examined and the neuropsychological impairments for the stage 2/3 group remained.

**Conclusions:**

The degree of neuropsychological impairment in young persons with mental disorders appears to discriminate those with attenuated syndromes from those with a discrete disorder, independent of diagnostic status and current symptoms. Our findings suggest that neuropsychological assessment is a critical aspect of clinical evaluation of young patients at the early stages of a major psychiatric illness.

## Background

There is recognition of the need for new clinical and research frameworks to enhance earlier intervention in young people with emerging major mental disorders (McGorry et al. 2009,2006; Fava et al. 2012; Hickie et al. 2013; Cosci and Fava 2013). To this end, the potential value of adapting clinical staging has been increasingly recognised (McGorry et al. 2006; Hickie et al. 2013b). These processes propose that it is possible to differentiate prodromal, sub-syndromal or ‘at-risk’ states from first major, acute or recurrent episodes, largely independent of diagnostic considerations. To date, the utility of clinical staging has been tested largely within those who present with psychotic symptoms. However, most young people who present for care with early but disabling forms of mental disorder have admixtures of anxiety, depressive or brief hypomanic or psychotic symptoms and are at risk of developing a broad range of adverse psychological, physical health and functional outcomes. For these individuals, we do not have diagnostic or predictive strategies to guide treatment selection or more individualised clinical practice.

Broader staging models have now been proposed for those young people who present with psychotic symptoms or features suggestive of a major mood disorder (McGorry et al. 2006; Hetrick et al. 2008). More recently, we have presented a detailed methodology (Hickie et al. 2013a) for the latest iteration of the model proposed by McGorry et al. (2006) for use in young people presenting with psychotic or mood syndromes. This latest version offers a more refined rating system, particularly with regards to stage 3 [see (Hickie et al. 2013a)]. Subsequently, we have conducted a number of key studies evaluating the relationships between these proposed early and later clinical stages and a range of potential biomarkers including structural brain imaging (Lagopoulos et al. 2012) and circadian parameters (Naismith et al. 2012). As cognitive impairment is one of the characteristic features of major mental disorders and as it can be reliably and objectively measured by formal neuropsychological testing, it represents one of the most important potential validators of our novel clinical staging framework. In this first report of a large cohort of young people, we test the proposition that different stages of illness (as an adjunctive rating to the traditional diagnostic categories) are associated with differential patterns of neuropsychological impairment.

## Methods

The study and consent procedure was approved by the University of Sydney Human Research Ethics Committee. All participants were determined by their referring clinician or mental health professional to have the mental and intellectual capacity to give written informed consent prior to participation in the study.

### Participants

One hundred and ninety four young people were recruited from specialised ambulatory care services (Youth Mental Health Clinic at the Brain & Mind Research Institute; and *headspace*, Campbelltown, Sydney, Australia (Scott et al. 2009; Scott et al. 2012)) for the assessment and early intervention of mental health problems. Importantly, the key inclusion criterion for this study were: (i) persons aged 18 to 30 years seeking professional help primarily for a depressive (unipolar or bipolar) and/or psychotic syndrome; and, (ii) willingness to participate in longitudinal research related to clinical and neurobiological outcomes (Lagopoulos et al. 2012; Hermens et al. 2011). Participants were asked to abstain from drug and alcohol use for 48 hours prior to testing.

Participants were excluded if they had insufficient fluency in the English language to participate in the neuropsychological assessment, were intellectually impaired (e.g. IQ < 70) or had current substance dependence. Comorbid or pre-existing childhood-onset conditions, such as ADHD and conduct disorder, as well as anxiety, alcohol or other substance misuse or autistic spectrum disorders were not exclusion criteria.

### Clinical staging

Our clinical staging model (Hickie et al. 2013a) builds on routine clinical assessment (though it may be assisted by ancillary investigations). Typically, a clinical stage is formally assigned at the end of the assessment phase. Such clinical assessment captures: (i) current major symptoms (severity, frequency, type); (ii) characteristic mental features; (iii) age of onset and clinical course of illness prior to presentation; (iv) previous “worst ever” symptoms and treatments including hospital admissions; (v) current level of risks of harm due to illness; (vi) previous suicide attempts or other at-risk behaviours; and, (vii) current (as compared with premorbid) levels of social, educational or employment functioning. Once this information is obtained and integrated, a clinical stage is then assigned according to sets of established criteria [see (Hickie et al. 2013a)]. It should be noted that in the most recent version of our model we stipulate that supporting instrumentation (e.g. socio-occupational and symptom rating scales) should be used as a guide and not as an absolute cut-off to determine stage. Similarly, biomarkers (i.e. from neuroimaging and neuropsychology) are subject to empirical research and are therefore not part of the stage assignation process.

As described in detail elsewhere (Hickie et al. 2013a), our staging model includes five discrete categories: stage 1a = ‘help-seeking’; stage 1b = ‘attenuated syndrome’; stage 2 = ‘discrete disorder’; stage 3 = ‘recurrent or persistent disorder’; and stage 4 = ‘severe, persistent and unremitting illness’. Importantly, entry to stage 2 is not simply analogous to, or defined by, meeting existing DSM or ICD criteria for a specific mood or psychotic disorder (the stage rating is adjunctive to the assignation of traditional DSM or ICD diagnoses). However, a key point of differentiation (and the focus of this study) occurs between the ‘attenuated syndrome’ stage (1b) and the onset of a more discrete disorder (stage 2). Thus only patients who were consensus rated at stage 1b, 2 or 3 by two senior psychiatrists (EMS and IBH) were included in this study. Stage 1b is assigned when the individual has developed specific symptoms of severe anxiety (including specific avoidant behaviour), moderate depression (associated with persistently depressed mood, anhedonia, suicidal ideation or thoughts of self-harm and/or some neurovegetative features), brief hypomania (less than 4 days duration during any specific episode) and/or brief psychotic phenomena (of brief duration only). Stage 2 is assigned when the individual displays a psychotic (i.e. a clear psychotic syndrome for more than a week), manic (i.e. manic syndrome (not just symptoms) for more than 4 days during a specific illness event) and/or severe depressive (i.e. psychomotor retardation, agitation, impaired cognitive function, severe circadian dysfunction, psychotic features, brief hypomanic periods, severe neurovegetative changes, pathological guilt and/or severe suicidality) episode. An individual with an anxiety disorder would be assigned to stage 2 if they have a concurrent, moderately severe depressive disorder, typically associated with marked agitation, fixed irrational beliefs, overvalued ideas or attenuated psychotic symptoms, or substantial and persistent substance misuse. Stage 3 is met if the discrete disorder persists over 12 months with poor or incomplete response to a reasonable course of treatment (i.e. of 3 months duration). Individuals who relapse to the full extent described in stage 2 are also assigned to stage 3. For details regarding the mixed syndromes and comorbid features within each stage assignation see (Hickie et al. 2013a).

A total of 194 patients were rated as stage 1b (n = 94), stage 2 (n = 69), or stage 3 (n = 31). In keeping with our previous research (Naismith et al. 2012; Lagopoulos et al. 2012) the last two stage-groups were combined (i.e. ‘stage 2/3’). The primary DSM-IV (APA 2000) diagnoses for those in stage 2/3 (n = 100) were as follows: n = 18 with a major depressive disorder; n = 25 with a bipolar disorder [bipolar I (n = 9); bipolar II (n = 16)] and n = 57 were diagnosed with a psychotic disorder [first-episode psychosis (n = 28); schizoaffective disorder (n = 11); schizophrenia (n = 17); psychotic disorder not otherwise specified (n = 1)].

### Clinical assessment

A trained research psychologist conducted a structured clinical interview to determine the nature and history of any mental health problems. Our ‘BMRI Structured Interview for Neurobiological Studies’ (Scott et al. 2013; Lee et al. 2013) initially obtains key demographic and clinical information, focussing on critical illness course variables (e.g. onset of symptoms, number of depressive, manic or psychotic episodes, hospitalisation, etc.). As a proxy measure for duration of illness, the age that each patient first engaged a mental health service was recorded. The interview then utilises established clinical scales including the 24-item Brief Psychiatric Rating Scale (BPRS) (Dingemans et al. 2013) and the 17-item Hamilton Depression Rating Scale (HDRS) (Hamilton 1967) to quantify general psychiatric and depressive symptoms at the time of assessment. The social and occupational functioning assessment scale (SOFAS) (Goldman et al. 1992) was also used as a rating of the patient’s functioning from 0 to 100, with lower scores indicating more severe impairment. Patients also completed self-report questionnaires that included the 10-item Kessler Psychological Distress Scale (K-10) (Kessler et al. 2002) to detect psychological distress.

### Neuropsychological assessment

Pre-morbid intelligence (‘predicted IQ’) was estimated on the basis of performance on the Wechsler Test of Adult Reading (Wechsler 2001). ‘Psychomotor speed’ was assessed using the Trail-Making Test (TMT), part A (TMT-A), with ‘mental flexibility’ assessed by part B (TMT-B) (Strauss et al. 2006). ‘Verbal learning’ and ‘verbal memory’ were assessed by the Rey Auditory Verbal Learning Test (RAVLT) (Strauss et al. 2006) sum of trial 1–5 (RAVLT sum) and 20-minute delayed recall (RAVLT A7) respectively. Finally, ‘verbal fluency’ was assessed by the letters subtest of the Controlled Oral Word Association Test (COWAT FAS) (Strauss et al. 2006). Participants also completed subtests from the Cambridge Neuropsychological Test Automated Battery (CANTAB) (Sahakian and Owen 1992). The CANTAB tests have the advantage of being largely non-verbal (i.e. language-independent, culture-free) and have been described in detail elsewhere (Sahakian and Owen 1992; Sweeney et al. 2000; Hermens et al. 2011). Four tasks were included for analysis in the current study: ‘sustained attention’, as indexed by the A prime (sensitivity to the target) measure of the Rapid Visual Information Processing task (RVP A), ‘working memory’ as indexed by the total span length from the Spatial Span task (SSP); ‘visuo-spatial learning and memory’ as indexed by the total adjusted errors score from the Paired Associate Learning task (PAL) and ‘set shifting’ was indexed by the total adjusted errors score from the Intra-Extra Dimensional task (IED errors).

### Statistical analyses

To control for the effects of age, neuropsychological variables were converted to ‘demographically corrected’ standardised scores (z-scores) using the following established norms: TMT (Tombaugh et al. 1998b); RAVLT (Rickert and Senior 1998); and COWAT FAS (Tombaugh et al. 1998a). Similarly, CANTAB z-scores, based on an internal normative database of the 3000 healthy volunteers (http://www.camcog.com), were calculated for each participant. Prior to analyses, outliers beyond ± 3.0 z-scores for each neuropsychological variable were curtailed to values of +3.0 or -3.0. There were no more than 7% of cases in any group with a z-score of beyond ±3.0 across variables. Differences in demographic, clinical and neuropsychological measures across the three groups were assessed using one-way ANOVA. Levene’s test was used to test for homogeneity of variance; Welch’s statistic was calculated, with corrected df and p-values reported where this assumption was violated. Scheffé’s tests were used to determine post-hoc pair-wise comparisons with the control group. Chi-squared test was used to compare the ratio of females to males across groups. Pearson’s correlations were used to test association between clinical and neuropsychological variables for patients only. Statistical analyses were performed using SPSS for Windows 20.0 and all significance levels were set at p<0.05.

## Results

As shown in Table 1, there were no differences amoung the three groups (i.e. Stage 1b, stage 2/3 and controls) in terms of their current age or predicted IQ. There was however a significant difference (p<.05) in the distribution of gender across the groups with the stage 2/3 group have the lowest proportion of females (43%) compared to the stage 1b group with the highest proportion (62%). There was also a significant main effect of group (p<.001) for years of education; post-hoc Scheffe’s tests confirmed that this was due to the controls having more formal education (at 14.8 ± 2.2 yrs) than the two patients groups – who did not differ from each other (see Table 1). There were similar, and somewhat expected, findings for the clinical measures. Social functioning (SOFAS), current depressive (HDRS) and general psychiatric (BPRS) symptoms as well as self-reported psychological distress (K-10) all showed a significant main effect at the group level (p<.001). This was primarily due to the controls being non-symptomatic (as expected), whereas the patient groups did not differ from each other aside from their SOFAS scores where the stage 2/3 group was rated lower than their stage 1b peers, by approximately 5 points (out of 100).

**Table 1:** **Mean scores (± standard deviation) for demographic and clinical variables between groups, tested by chi-square or ANOVA**

	***Stage 1b (n = 94)***	***Stage 2/3 (n = 100)***	***Controls (n = 50)***	***Significance Test [p]***	***Post hoc***		
					1b v 2/3	1b v Ctrl	2/3 v Ctrl
Females, n (%)	58 (62%)	43 (43%)	29 (58%)	χ^2^ (2, 244) = 7.4 [.025]			
Age, years	22.2 ± 3.2	23.0 ± 3.3	23.0 ± 2.7	F (2, 243) = 2.2 [.118]			
Age of onset, years	15.4 ± 3.3	17.6 ± 4.9	n/a	F (1, 160.8) = 12.7 [.000]			
Predicted IQ	103.0 ± 8.5	103.2 ± 10.8	106.0 ± 7.8	F (2, 242) = 1.9 [.148]			
Education, years	12.8 ± 2.1	13.1 ± 2.4	14.8 ± 2.2	F (2, 243) = 13.7 [.000]		***	***
SOFAS	61.4 ± 11.3	56.4 ± 12.5	92.0 ± 3.2	F (2, 143.9) = 557.5 [.000]	**	***	***
HDRS total	12.9 ± 6.5	12.5 ± 8.5	2.0 ± 2.2	F (2, 150.2) = 149.6 [.000]		***	***
BPRS total	40.5 ± 9.6	42.7 ± 12.3	26.5 ± 2.7	F (2, 147.4) = 146.2 [.000]		***	***
K10 total	28.1 ± 7.7	26.0 ± 9.1	15.4 ± 5.1	F (2, 135.5) = 72.4 [.000]		***	***

*Note:* Significance levels for each Scheffé’s post-hoc comparison are depicted by: *** = p<.001; ** = p<.01.

**Figure 1:**
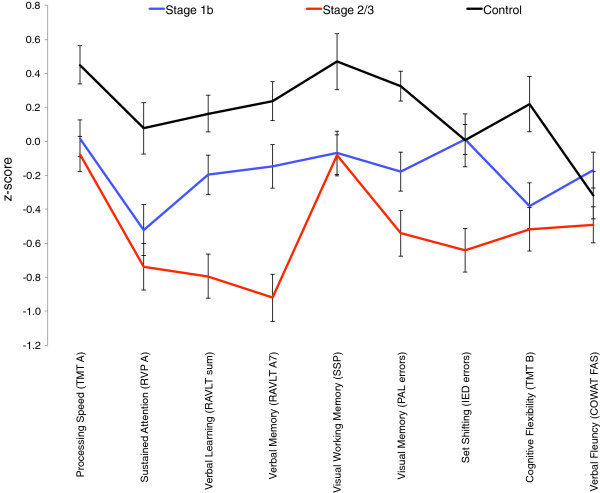
**Profile of** ***z-*** **scores (with standard error bars) for neuropsychological measures across the stage 1b (n = 94), stage 2/3 (n = 100) and control (n = 50) groups.**

The neuropsychological profiles (mean z-scores) for all three groups are depicted in Figure 1 and the corresponding ANOVAs and post-hoc tests are summarised in Table 2. With the exception of verbal fluency (COWAT FAS), the control group showed a normal profile of neuropsychological function with all variables averaging between 0.0 and 0.5 standardised scores. In contrast, the stage 2/3 group showed the worst profile with neuropsychological z-scores ranging between 0.0 and -1.0; the stage 1b group showed an intermediate profile (see Figure 1). The differences in these three profiles was confirmed by the ANOVA’s which showed a significant (at least p<.05) main effect of group for all but one variable. The lack of a difference in verbal fluency is consistent with the lack of differences in the premorbid IQ measure (which is based on a verbal IQ score). Post-hoc Scheffe’s tests revealed that for the remaining eight neuropsychological variables (i.e. not including verbal fluency) the stage 2/3 group performed significantly worse than controls. As compared to the stage 1b group, stage 2 patients were worse on three variables: verbal learning (RAVLT sum), verbal memory (RAVLT A7) and set-shifting (IED errors). Interestingly, for the remaining five variables, the stage 1b group was significantly worse than controls but no different (statistically) to the stage 2 group (see final three columns in Table 2). Follow-up ANCOVAs revealed that all of the eight neuropsychological variables remained significant after controlling for gender.

**Table 2:** **Mean z-scores (± standard deviation) for neuropsychological variables between groups, tested by ANOVA**

	***Stage 1b (n = 94)***	***Stage 2/3 (n = 100)***	***Controls (n = 50)***	***Significance Test [p]***	***Post hoc***		
					1b v 2/3	1b v Ctrl	2/3 v Ctrl
TMT A	0.02 ± 1.04	-0.08 ± 1.03	0.45 ± 0.79	F (2, 140.2) = 6.5 [.002]		*	*
RVP A	-0.52 ± 1.34	-0.74 ± 1.27	0.08 ± 1.05	F (2, 210) = 6.6 [.002]		*	**
RAVLT sum	-0.20 ± 1.12	-0.79 ± 1.30	0.16 ± 0.76	F (2, 148.6) = 15.9 [.000]	**		***
RAVLT A7	-0.15 ± 1.23	-0.92 ± 1.40	0.24 ± 0.81	F (2, 148.2) = 20.3 [.000]	***		***
SSP	-0.07 ± 1.18	-0.08 ± 1.11	0.47 ± 1.13	F (2, 217) = 4.2 [.017]		*	***
PAL errors	-0.18 ± 1.05	-0.54 ± 1.25	0.33 ± 0.61	F (2, 141.8) = 16.0 [.000]		*	***
IED errors	0.01 ± 0.82	-0.64 ± 1.21	0.01 ± 1.08	F (2, 116.3) = 9.3 [.000]	***		**
TMT B	-0.38 ± 1.29	-0.52 ± 1.28	0.22 ± 1.12	F (2, 230) = 5.8 [.004]		*	**
COWAT (FAS)	-0.17 ± 0.99	-0.49 ± 1.04	-0.32 ± 0.14	F (2, 230) = 2.4 [.091]			

*Note:* Significance levels for each Scheffe post-hoc comparison are depicted by: *** = p<.001; ** = p<.01; * = p<.05.

As shown in Table 3, the proportion of patients who were currently medicated with an anti-depressant was comparable in the stage 1b (54%) and stage 2/3 (45%) groups. However, there were three times more cases in stage 2/3 who were currently taking an anti-psychotic and/or a mood stabiliser; whereas stage 1b patients were six times more likely to not be taking a major psychotropic medication at the time of testing (see Table 3). While there were no significant associations between the symptom measures (HDRS; BPRS) and neuropsychological variables for the entire patient sample, there were significant Pearson’s correlations for the stage 1b group only. These patients (stage 1b) showed a significant negative correlation between TMT-B and both HDRS total [r(91)= -0.30, p<.01] and BPRS total [r(90)= -0.28, p<.01] scores. Similarly, the stage 1b groups showed significant correlations between RVP A and both HDRS total [r(78)= -0.23, p<.05] and BPRS total [r(77)= -0.28, p<.05] scores. In all correlations, poorer performance was associated with worse symptoms. Of note, the stage 2/3 group showed no significant correlations between these variables.

**Table 3:** **Cross-tabulation of stage group by medication category**

***Current Medication***		***Stage 1b (n = 94)***	***Stage 2/3 (n = 100)***
NIL	Count	25	4
	%	27%	4%
Any Anti-Depressant	Count	51	44
	%	54%	44%
Any Anti-Psychotic	Count	26	78
	%	28%	78%
Any Mood Stabiliser	Count	7	25
	%	7%	25%

**Figure 2:**
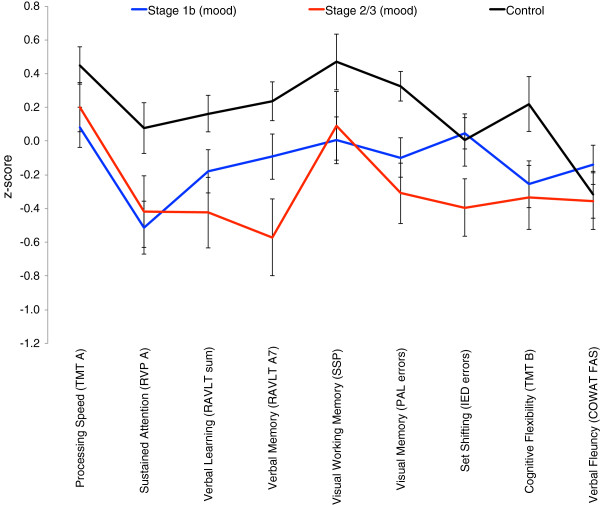
**Profile of** ***z-*** **scores (with standard error bars) for neuropsychological measures across the mood syndrome/disorder subset at stage 1b (n = 79) and stage 2/3 (n = 41), versus control (n = 50) groups.**

In order to address potential confounds created by ‘diagnosis’, neuropsychological profiles for those identified as having a mood syndrome or disorder but not psychosis were also examined. Figure 2 shows the neuropsychological profiles for subsamples of the stage 1b (N = 79) and stage 2/3 (N = 41) patients. As compared to the same control group, these subsamples show very similar profiles as seen in the stage-groups which included patients with psychosis with significant (p<.05) main effects of group for five neuropsychological variables (RVP A; RAVLT sum; RAVLT A7; PAL errors and TMT-B). While the magnitude of impairment was less severe, in the stage 2/3 group the verbal learning (RAVLT sum), verbal memory (RAVLT A7) and visual memory (PAL errors) remained significantly (p<.05) worse than controls. Whereas the stage 1b group only differed significantly (p<.05) from controls in RVP A (see Figure 2).

## Discussion

This study identified distinct neuropsychological profiles that distinguished those young people with ‘attenuated syndromes’ from those with a discrete or persistent disorder, independent of other diagnostic considerations. As expected, those in the later stages showed the most impaired neuropsychological profile with the attenuated syndrome patients showing an intermediate profile compared to controls (as well as the standardised ‘norm’). These neuropsychological findings are especially important given the lack of differences between the patient groups in terms of their overall current symptoms and levels of distress. These findings provide further important validation of our clinical staging model, particularly with respect to the notion that the change from stage 1b to stage 2 and 3 represents a ‘key point of differentiation’ (Hickie et al. 2013a).

The findings presented here are consistent with our other studies showing a similar demarcation in both neuroimaging (Lagopoulos et al. 2012) and circadian (Naismith et al. 2012) measures. In the former study, there were frontal grey matter volume differences between the stage 1b and stage 2/3 groups, suggesting a major transition point (Lagopoulos et al. 2012). In the latter study, stage 2/3 patients, but not stage 1b patients, showed a disruption in a circadian system marker (reduced melatonin secretion) which was associated with less subjective sleepiness and poorer performance in a memory task (Naismith et al. 2012). In relation to neuropsychological profiles, there is very little other literature to compare our results to. While numerous studies have described the neuropsychological profiles of prodromal or ‘ultra-high risk’ states for psychosis there are, to our knowledge, no studies that have included young patients with unipolar and/or bipolar illnesses. This may be a critical oversight, given evidence that affective and psychotic disorders probably represent different combinations of the same continuously distributed dimensions of symptoms, particularly at early stages (Hafner et al. 2008). Importantly, our clinical staging model (Hickie et al. 2013a) maintains that there is inherent heterogeneity of cases within each clinical stage and that more detailed profiling (using syndromal, psychological and neurobiological measures) is required to better understand the key underlying factors that cause patients to express a discrete disorder or not (that is, despite being similar in age and current symptomatology).

Our samples are representative of young help-seeking outpatients with admixtures of depressive, (hypo)manic and psychotic symptoms. However, there is some evidence to suggest that those with psychotic spectrum illness show the most marked neuropsychological impairments at various ages (Quraishi and Frangou 2002). Therefore we also examined the neuropsychological profiles of only those with a mood syndrome or disorder and our results confirm that such patients showed a similar overall pattern as the larger sample (with psychosis included), albeit to a lesser degree. Critically, the two key neuropsychological variables (i.e. verbal memory and set-shifting, an aspect of executive functioning) remained significantly different across the clinical-stage groups (and markedly reduced in the stage 2/3 patients). Separate lines of research have shown that cognitive decline in the form of verbal memory and executive function deficits is characteristic of (and often precedes) the early stages of both affective (Burt et al. 1995) and psychotic (Brewer et al. 2005; Seidman et al. 2010) disorders. Similarly, there are several studies showing that impaired neuropsychological function (particularly with regards to memory and executive function) in early stage young patients with mental disorders predicts longer-term poor (typically functional) outcomes (Bodnar et al. 2008; Seidman et al. 2010). Thus, it is becoming increasingly important to identify the best neuropsychological markers for early intervention. This is particularly warranted given that pharmacological (e.g. antidepressant) (Sheline et al. 2003) and non-pharmacological (e.g. cognitive training) (Naismith et al. 2010) strategies may offer neuroprotection against further cognitive damage (Simon et al. 2007).

This study has limitations. Firstly, the cross-sectional design impacts any conclusions about which neuropsychological variables reflect trait versus state aspects of these stages of illness. The presence of some significant associations between the sustained attention or cognitive flexibility measures and current depressive or general psychiatric symptoms (in the stage 1b group) suggests that at least some aspects of executive functioning may be modulated by an individuals state. Clearly longitudinal studies would provide very important information about such trait versus state aspects. Secondly, we did not control for any potential effects of psychotropic medication. Although we opted to assess these young patients under ‘treatment as usual’ conditions, the real impact that such medications have on neuropsychological function is unknown. Given the early stage of illness it is unlikely that the current medications afforded any neuroprotection, but rather offered some amelioration of affective and/or psychotic symptoms. While the clinical-stage groups did not differ in the prevalence of current antidepressant treatment there were differences in the frequency of antipsychotics and mood stabilisers. While there is good evidence to show that the former have very little direct effects on cognition, particularly at early stages in the course of treatment, there is less known about these effects from the latter. Thirdly, the control group in this study were more educated than the patient groups. Despite this, all three groups were matched in their predicted IQ and the standardised scores for each neuropsychological variable were adjusted for age and years of education. Fourthly, while the lack of a significant difference in age among groups was helpful in evaluating the differences in neuropsychological function it may also limit the generalizability of our findings. In our previous study (Scott et al. 2012), utilising a much larger (N = 1260), albeit younger (i.e. 12 to 25 years of age) sample of patients (accessing the same services as those in the current study), we reported age differences among the three stage groups (stage 1b = 17.4 ± 3.4 years; stage 2 = 18.7 ± 3.2 years; stage 3 = 20.3 ± 3.4 years). Given the different age range in the current study (in particular the minimum age of 18 years) these findings may only represent young adults at various stages of illness; future studies should include younger patients (despite the limitations in normative data and valid neuropsychological subtests for younger subjects). Another limitation may be the significant differences among groups in terms of the proportions of females-to-males. Just over two-thirds (62%) of those in the stage 1b group were female, compared to the lower proportion of females (43%) in the stage 2/3 group. These ratios are quite different to those in our larger, younger cohort (Scott et al. 2012) with 47% and 54% females in stage 1b versus stage 2/3, respectively. Although our statistical analyses attempted to control for the effects gender, our findings should be treated with some caution until future studies with larger sample sizes (and presumably more equal proportions of the genders) are conducted. Finally, as highlighted in a recent systematic review (Cosci and Fava 2013) there are numerous variations of staging models for mental disorders. In their distillation of this literature, Cosci and Fava (2013) propose separate models for a range of disorders (including schizophrenia, unipolar depression, bipolar and alcohol use disorders). Thus, it is important to recognise the distinctions between the model investigated in this current study and others in the literature. Comprehensive longitudinal research will help to determine the utility of staging within single disorders (see (Cosci and Fava 2013)) versus staging across a range of syndromes (Hickie et al. 2013; Hickie et al. 2013a).

## Conclusions

In conclusion, this study is the first of its kind and shows that there is a neuropsychological point of differentiation in young persons with an attenuated syndrome as compared to those with a discrete or persistent disorder. While those in the latter group show impairments in memory and executive measures that are consistent with the literature, the ‘intermediate’ profile seen in the attenuated syndrome patients suggest that they are on a similar neuropsychological trajectory despite current symptoms and, possibly, current treatment. These findings add strength to our clinical staging model and support our findings in other neurobiological measures (Naismith et al. 2012; Lagopoulos et al. 2012). Furthermore, these findings suggest that neuropsychological assessment is a critical aspect of clinical evaluation of young patients at the early stages of a major psychiatric illness.

## Authors’ information

EMS is the Clinical Director of the *headspace* clinics at the Brain & Mind Research Institute. IBH was a director of *headspace*: the national youth mental health foundation until January 2012. He is the executive director of the Brain & Mind Research Institute, which operates two early-intervention youth services under contract to *headspace*. He is a member of the new Australian National Mental Health commission and was previously the CEO of beyondblue: the national depression initiative.

## Abbreviations

ANOVA: Analysis of variance
BPRS: Brief psychiatric rating scale
COWAT (FAS): Controlled oral word association test – letters subtest
DSM: Diagnostic and statistical manual of mental disorders
HDRS: Hamilton depression rating scale
ICD: International classification of diseases
IED errors: Intra-extra dimensional, total errors
K10: Kessler-10
PAL errors: Paired associates learning – total errors
RAVLT sum: Rey auditory verbal learning test - sum of five learning trials
RAVLT A7: Rey auditory verbal learning test - delayed recall
RVP A: Rapid visual processing - correct responding
SOFAS: Social and occupational functioning assessment scale
SPSS: Statistical package for the social Sciences
SSP: Spatial span
TMT-A: Trail-making test - part A
TMT B: Trail making test - part B.
